# Exploring the pharmacological mechanisms of Shuanghuanglian against T-cell acute lymphoblastic leukaemia through network pharmacology combined with molecular docking and experimental validation

**DOI:** 10.1080/13880209.2023.2168703

**Published:** 2023-01-19

**Authors:** You Yang, Yan Yang, Yunfu Shen, Jing Liu, Yan Zeng, Chengming Wei, Chunyan Liu, Yansha Pan, Qulian Guo, Fangfang Zhong, Ling Guo, Wenjun Liu

**Affiliations:** a Department of Pediatrics (Children Hematological Oncology), Birth Defects and Childhood Hematological Oncology Laboratory, The Affiliated Hospital of Southwest Medical University, Sichuan Clinical Research Center for Birth Defects, Luzhou, Sichuan, China; b State Key Laboratory of Quality Research in Chinese Medicine, Macau University of Science and Technology, Macau, China

**Keywords:** T-ALL, NOTCH1, apoptosis

## Abstract

**Context:**

Due to the poor prognosis of T-cell acute lymphoblastic leukaemia (T-ALL), there is an urgent need to identify safer and more cost-effective drugs.

**Objective:**

This study evaluated the antitumour activity of Shuanghuanglian (SHL) on T-ALL cells and elucidated the mechanism.

**Materials and methods:**

Jurkat and Molt4 cells were treated with SHL (0.1, 0.2 and 0.4 mg/mL) for 24 and 48 h. The controls were treated with RPMI 1640 containing 10% foetal bovine serum. Cell viability was evaluated through Cell Counting Kit-8 assay. Patterns of death and signalling pathway alterations caused by SHL were identified by network pharmacology combined with GO enrichment analysis and then were verified by Hoechst 33342 staining, Annexin V-FITC/PI staining and Western blotting. Interactions of the active ingredients with targets were analysed by molecular docking.

**Results:**

The IC_50_ values of SHL in Jurkat and Molt4 cells were 0.30 ± 0.10 and 0.48 ± 0.07 mg/mL, respectively, at 24 h and 0.27 ± 0.05 and 0.30 ± 0.03 mg/mL at 48 h. In T-ALL, 117 target genes of SHL were mainly enriched in the apoptosis and NOTCH signalling pathways. SHL induced apoptosis was confirmed by Hoechst 33342 staining and flow cytometry. The protein levels of cleaved caspase-7 and cleaved PARP were significantly increased but those of cleaved NOTCH1 and MYC were reduced. The active ingredients of SHL can interact with γ-secretase.

**Discussion and conclusions:** SHL induces apoptosis in T-ALL cells *via* the NOTCH1-MYC pathway and may be a potential drug for the treatment of T-ALL.

## Introduction

T-cell acute lymphoblastic leukaemia (T-ALL) is an aggressive blood disorder caused by the continuous clonal proliferation of T-cell precursors that develop in the thymus. The incidence is approximately 25% in children and 15% in adults, and it is twice as high in males as in females (Bayón-Calderón et al. 2020; Lato et al. 2021). T-ALL patients typically present with high-risk clinical features, including an elevated leukocyte count, an older age and extramedullary disease. Compared with patients with B-ALL, T-ALL patients tend to have less tumour cell clearance after initial treatment and are prone to relapse during active treatment, thus leading to a poor prognosis (Winter et al. 2018; Malard and Mohty 2020). In addition, great challenges remain, as up to one in five children still exhibit drug resistance, and relapse and treatment-related mortality are concerns (Wang et al. 2018; Teachey and Connor 2020).

Among the various methods of cancer treatment, traditional Chinese medicine (TCM) and natural products have outstanding anticancer effects due to their unique advantages of high efficiency and few side effects (Liu et al. 2020). However, TCMs with high efficacy and few side effects are lacking in the clinical treatment of T-ALL at present. Identifying more effective anti-T-ALL drugs is an urgent need, and TCM may provide novel insight. Therefore, we screened the potential anti-leukaemia activity of TCMs in our TCM library using T-ALL cell models. Interestingly, the lyophilized powder used for injection of SHL, which has been used for many years in the traditional treatment of respiratory infections and consists of honeysuckle, forsythia, and skullcap, was found to inhibit T-ALL cell proliferation (Xu et al. 2019; Yang et al. 2021). However, as SHL is a well-known TCM, recent reports on SHL have mainly focussed on its antibacterial and antiviral effects (Xu et al. 2019). It was reported that SHL inhibited the replication of SARS-CoV-2 by inhibiting SARS-CoV-2 3CLpro (Su et al. 2020). Ma et al. (2017) reported that SHL can be used to successfully treat children with mycoplasma pneumonia when combined with azithromycin. SHL could play an antiviral role against H1N1 virus through TNF signalling pathways (Zhang et al. 2021). SHL has a long history in the treatment of respiratory tract infections in China (Xu et al. 2019). However, information on the anticancer effect of SHL is unavailable. Compared with SHL, common chemotherapeutic drugs for T-ALL have more side effects, such as myelosuppression and severe infection (Asselin et al. 2016; Tang et al. 2016; Winter et al. 2018; Hayashi et al. 2020). Therefore, it is particularly necessary to explore the underlying anti-T-ALL mechanism of SHL, which may provide new insight for the clinical treatment of T-ALL.

With the advancement of systems biology and bioinformatics approaches, the concept of network pharmacology was proposed (Guo et al. 2022). Unlike the traditional dogma of ‘one disease, one target, one drug’, the complexity of interactions among different biological systems, drugs and diseases can be analysed and demonstrated from the network perspective using network pharmacology (Casas et al. 2019). Therefore, network pharmacology has been widely used to explore the mechanisms of TCMs (Zheng et al. 2018; Xu et al. 2020). In this study, network pharmacology combined with molecular docking and experimental validation was employed to explore the potential mechanism of SHL in the treatment of T-ALL.

## Materials and methods

### Chemicals and reagents

The lyophilized powder used for injection of SHL (lyophilized powder) was obtained from a hospital and was manufactured by Harbin Pharmaceutical Second Factory Chinese Medicine Group Co., Ltd (Batch No.: Z10960058). A FITC Annexin V Apoptosis Detection Kit was purchased from BD Biosciences Pharmingen (San Diego, CA); Hoechst 33342 was purchased from Solarbio (Beijing, China). A CCK-8 Kit was purchased from Beyotime (Shanghai, China). Antibodies against β-actin, cleaved NOTCH1, cleaved caspase-7, cleaved PARP, and c-Myc and anti-rabbit IgG-HRP were purchased from Cell Signaling Technology (Danvers, MA, USA). Dexamethasone sodium phosphate for injection was obtained from a hospital and was manufactured by Shanghai Hyundai Hassen (Shangqiu) Pharmaceutical Co., Ltd. (Batch No: H41021255).

### Cell culture

The human T-ALL cell lines Jurkat and Molt4 were purchased from the Cell Bank of Shanghai Institute of Biochemistry and Cell Biology. The culture medium was RPMI 1640 (Gibco, Beijing, China) supplemented with foetal bovine serum, penicillin (100 IU/mL) and streptomycin (100 μg/mL) (Gibco, Grand Island, NY, USA)

### Cell viability assay

Cell viability was determined by a CCK-8 Kit (Beyotime, Shanghai, China) (Wang et al. 2018). Briefly, 2.0 × 10^4^ Jurkat/Molt4 cells in medium (100 μL) were seeded in a 96-well culture plate. According to a previous study (Yang et al. 2021), different concentrations of SHLs (0, 0.1, 0.2, 0.3, 0.4, 0.5 and 0.6 mg/mL) were added for 24 and 48 h of treatment. The samples were incubated with 10 μL of CCK-8 solution for another 4 h at 37 °C and a microplate reader (Thermo Scientific Multiskan Sky, MA, USA) was used to measure the absorbance at 450 nm. Cell viability (CV) was calculated according to the manufacturer’s instructions.

$$\left(CV\%\right)=\frac{A_{\mathrm{Treament}}}{A_{\mathrm{Control}}}\times 100\%$$

The half-maximal inhibitory concentration (IC_50_) was determined using GraphPad Prism 8.0 software (San Diego, CA, USA).

### Apoptosis assay

Based on the IC_50_ values obtained in the cell viability assay and a previous study (Yang et al. 2021), Jurkat/Molt4 cells were seeded in a six-well culture plate at a density of 2.0 × 10^5^ cells/well and treated with 0, 0.1, 0.2 and 0.4 mg/mL SHLs for 24 h. To demonstrate that the rate of apoptosis induced by SHL could be rescued by treatment with an apoptosis inhibitor, Jurkat/Molt4 cells were seeded in a six-well culture plate at same density and treated with SHL (0 and 0.4 mg/mL), Z-VAD-FMK (20 μM) or the combination SHL (0.4 mg/mL) and Z-VAD-FMK (20 μM) for 24 h. Benzyloxycarbonyl-Val-Ala-Asp-fluoromethyl ketone (Z-VAD-FMK) is a pancaspase inhibitor (Park et al. 2019). Apoptosis rates were measured by using a FITC Annexin V Apoptosis Detection Kit (BD Biosciences Pharmingen, San Diego, CA) (Wong et al. 2019). Treated cells were harvested and stained with annexin V-FITC and propidium iodide. After incubation at RT in the dark for 15 min, the mixtures were analysed by flow cytometry (BD FACSVerse, San Diego, CA, USA) and CellQuest software (BD, FACSuite). The cells were collected and stained with Hoechst 33342 (Solarbio, Beijing, China) for 30 min at room temperature. Fluorescence microscopy (Olympus IX73, Tokyo, Japan) was used to monitor the cells.

### Western blot analysis

Western blot analysis was performed according to previously described methods (Wang et al. 2021). Briefly, treated cells were lysed using RIPA buffer. After extraction, total protein was quantified by a Bradford dye binding assay with normalization to bovine serum albumin. Then, the extracted proteins were separated by sodium dodecyl sulphate–polyacrylamide gel electrophoresis and transferred to PVDF membranes (Merck Millipore, Darmstadt, Germany). The membranes were then incubated with specific primary antibodies at 4 °C overnight. After washing three times with PBST buffer, the membranes were incubated with the corresponding secondary antibody for 1 h at RT. An enhanced chemiluminescence system (Beijing Si’an Biotechnology Co., Ltd., Beijing, China) was used for imaging. Band densities were quantified by using ImageJ software (Version 1.53a, National Institutes of Health, Bethesda, MD, USA).

### Identification of chemical components of SHL and screening of active compounds

SHL consists of honeysuckle, skullcap and forsythia. The compounds of each herb in SHL were obtained through the Traditional Chinese Medicine Systems Pharmacology Database and Analysis Platform (TCMSP, https://old.tcmsp-e.com/tcmsp.php) (Ru et al. 2014). Compounds in SHL with oral bioavailability (OB) ≥ 30% and drug similarity (DL) ≥ 0.18 (Jia et al. 2020) were identified as pharmacologically active compounds in this study based on the absorption, distribution, metabolism and excretion (ADME) characteristics of the drugs in humans.

### Prediction of potential targets of SHL in T-ALL treatment

Information on the targets of active ingredients in SHL was acquired from the TCMSP and DrugBank databases (Wishart et al. 2008). Information on T-ALL-related targets was acquired from databases, i.e., the GeneCards database (https://www.genecards.org/) (Rebhan et al. 1997), and the Online Mendelian Inheritance in Man (OMIM) database (https://www.omim.org/) (Hamosh et al. 2005), using ‘T-cell acute lymphoblastic leukemia’ as the keyword. The targets of the active compounds of SHL and T-ALL-related targets were intersected to identify potential targets of SHL in the treatment of T-ALL.

### Gene ontology (GO) enrichment analysis

The potential targets of SHL for in T-ALL treatment were identified using the clusterProfiler, org.Hs.eg.db, enrichplot and ggplot2 R packages for GO enrichment analysis, annotation and visualization (Guo et al. 2022). A threshold of *p* < 0.05 was used to identify key GO terms.

### Molecular docking simulations

Molecular docking simulations of γ-secretase and active ingredients of SHL were performed by SeeSAR (https://www.biosolveit.de/SeeSAR/) (Chen and Zhong 2020). Affinities, torsion quality, intramolecular clash and intermolecular clash values were calculated to assess interactions between the protein and the drugs. Binding affinity values in SeeSAR are reported as inhibition constant (Ki) versus molar units. All drugs with an estimated lower bound binding affinity of less than 100 μM were chosen to for a screen with 70 poses. ProteinsPlus was used to visualize 2 D structures of γ-secretase bound to ligands (Zentrum für Bioinformatik: Universität Hamburg-Proteins Plus Server).

### Statistical analysis

Data were presented as mean ± standard deviation (SD) values. One-way analysis of variance (ANOVA) was used for comparisons among multiple groups. *p* < 0.05 was considered statistically significant. Analysis and graphing were performed using GraphPad Prism 8.0 software (San Diego, CA, USA).

## Results

### SHL inhibited T-ALL cells (Jurkat, Molt4) proliferation

**Figure 1.**
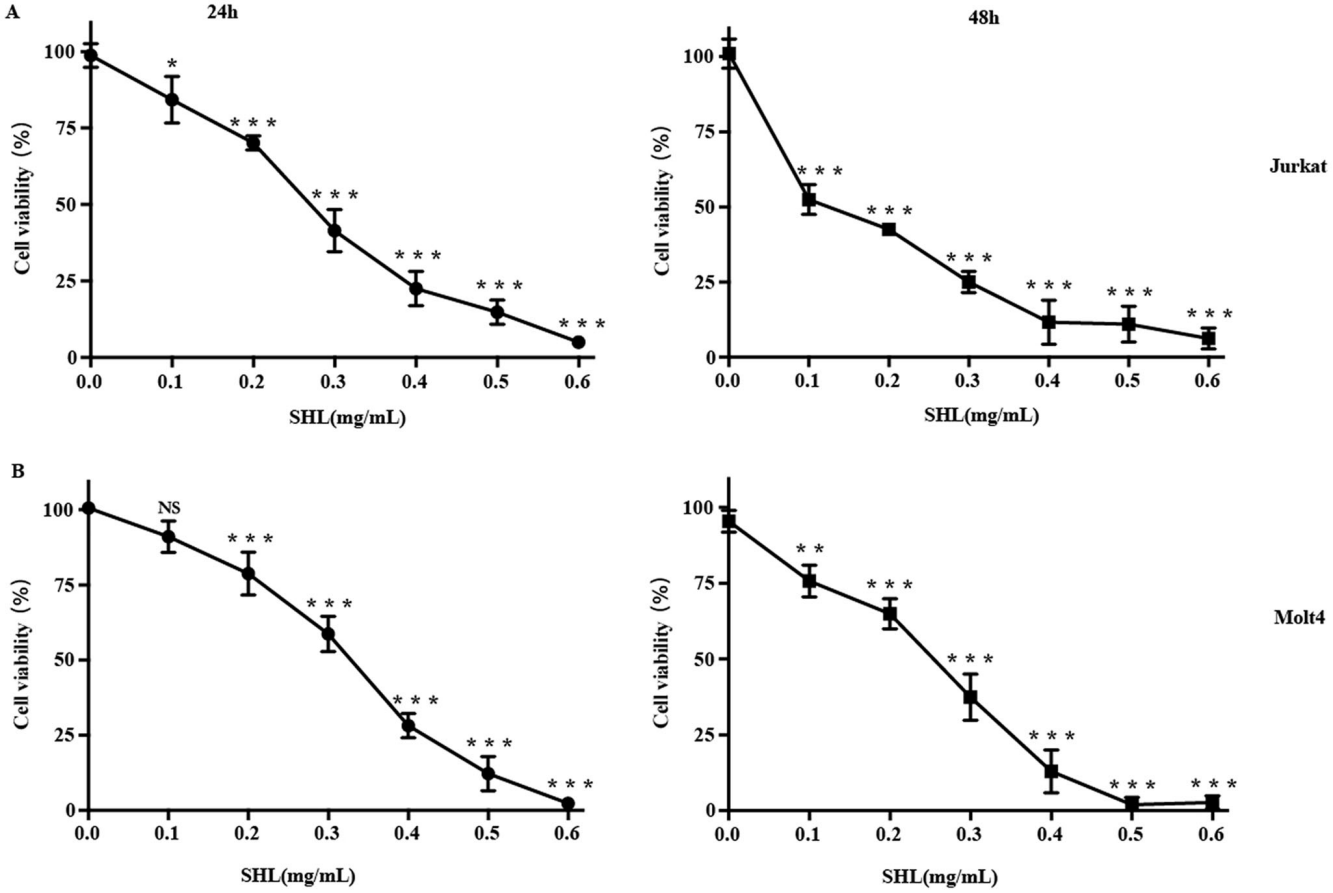
Cytotoxicity of SHL in T-ALL cells (Jurkat and Molt4). (A) Cytotoxicity of SHL in Jurkat cells (24 h, 48 h). (B) Cytotoxicity of SHL in Molt4 cells (24 h, 48 h). All data are shown as the means ± SD. *p < 0.05 compared with the corresponding controls. **p < 0.01 compared with the corresponding controls. ***p < 0.001 compared with the corresponding controls.

First, the effect of SHL on the viability of T-ALL cells (Jurkat and Molt4) was evaluated by a CCK-8 assay. Dexamethasone (DEX), a common chemotherapeutic agent for T-ALL, was selected as a positive control (Figure S4). Cells were treated with SHL at 0.1, 0.2, 0.3, 0.4, 0.5 and 0.6 mg/mL for 24 h and 48 h. The results showed that SHL inhibited the proliferation of T-ALL cells (Jurkat and Molt4) and that cell viability decreased dramatically as the SHL concentration increased (Figure 1(A,B)). The IC_50_ values in Jurkat and Molt4 cells were 0.30 ± 0.10 and 0.48 ± 0.07 mg/mL, respectively, at 24 h and 0.27 ± 0.05 and 0.30 ± 0.03 mg/mL at 48 h. These results indicated that SHL reduced the viability of both cell lines, suggesting that SHL may be a potential therapeutic agent. Based on the cell viability assay results, SHL concentrations of 0, 0.1, 0.2 and 0.4 mg/mL were selected for the subsequent experiments.

### Exploration of the mechanism by which SHL inhibited the proliferation and promoted the death of T-ALL cells

**Figure 2.**
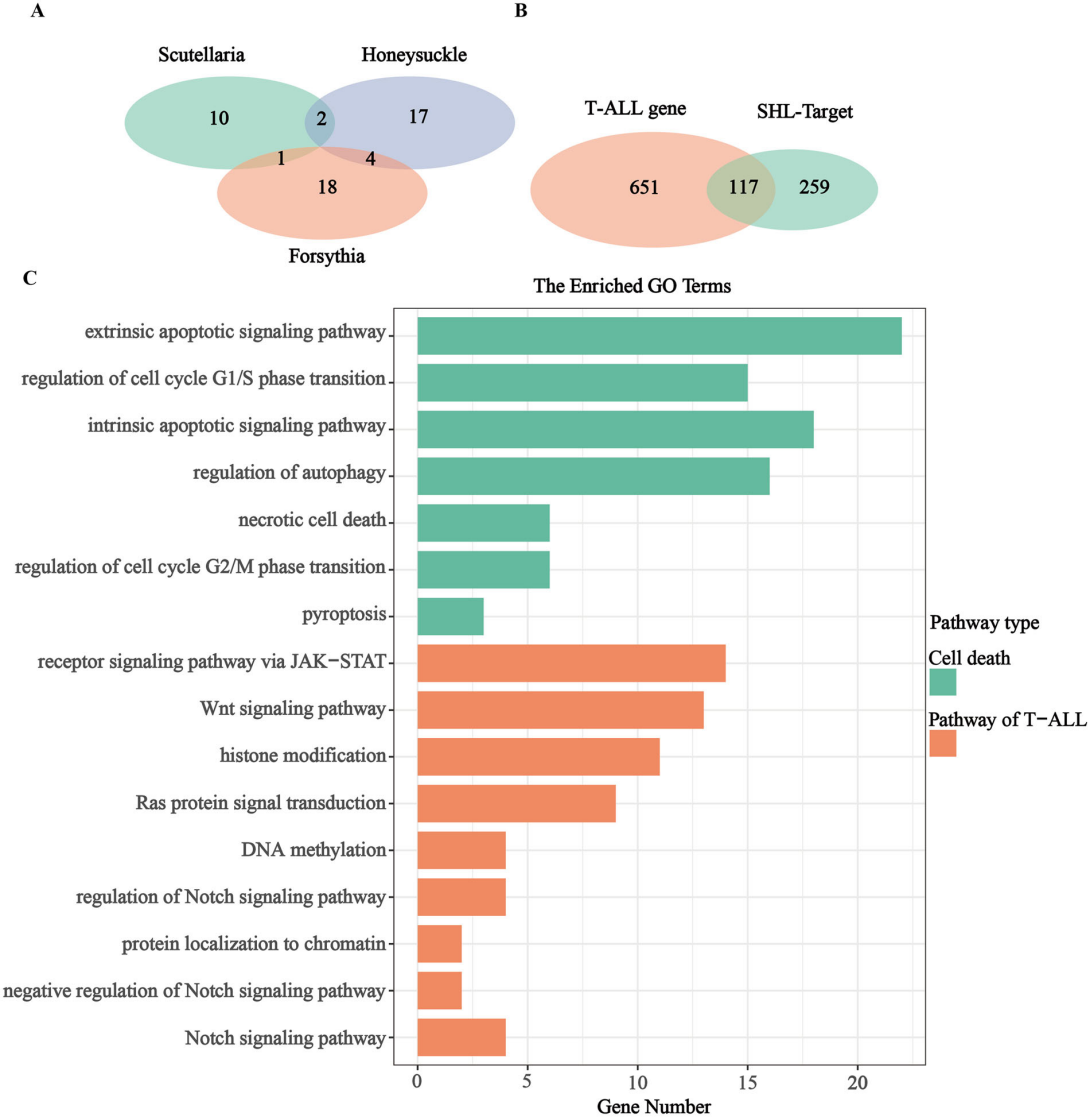
Exploration of the mechanism by which SHL inhibits the proliferation and promotes the death of T-ALL cells. (A) Venn diagram of the active ingredients of SHL; (B) Venn diagram of T-ALL and SHL targets; (C) GO enrichment analysis of the overlapping target genes in T-ALL and SHL.

SHL is a combination of three traditional Chinese medicines, including skullcap, honeysuckle and forsythia. To explore why SHL inhibits the proliferation and promotes cell death of T-ALL cells, the main active ingredients of the three herbs and their targets were searched in the TCMSP database. Skullcap, honeysuckle and forsythia contain 13, 23 and 23 active ingredients respectively (Figure 2(A)). A total of 376 target genes among the active ingredients were identified. Next, the target genes of T-ALL were identified through the GeneCards database and OMIM database. A total of 711 target genes were identified. There were a total of 117 overlapping target genes between T-ALL and SHL (Figure 2(B)).

After GO enrichment analysis, these 117 target genes were found to be mainly enriched in cell death pathways, including the extrinsic and intrinsic apoptotic signalling pathways; the regulation of G1/S- and G2/M- phase transition pathways, the regulation of autophagy pathway; the necrotic cell death pathway; and the pyroptosis pathway (Figure 2(C)). In addition, these 117 genes were enriched in the pathways commonly related to T-ALL, such as the regulation of NOTCH signalling pathway, NOTCH signalling pathway, negative regulation of NOTCH signalling pathway (Adrián et al. 2018; Fattizzo et al. 2020), Ras protein signal transduction pathway, Wnt signalling pathway, receptor signalling pathway *via* JAK-STAT, DNA methylation pathway, histone modification pathway and protein localization to chromatin pathway. According to the enrichment results (Figure 2(C)), it was not difficult to conclude that the mechanisms of SHL-induced T-ALL cell death were mainly centred on cell cycle control, necrosis, apoptosis, autophagy and pyroptosis. Among these pathways, apoptosis was dominant. Therefore, we speculated that SHL mainly induces T-ALL cell death through apoptosis promotion. Furthermore, one-third of the enriched common signalling pathways in T-ALL were involved in NOTCH signalling. Thus, we hypothesized that mechanistically, SHL induces T-ALL cell death *via* regulation of the NOTCH signalling pathway.

### SHL promoted T-ALL cell (Jurkat and Molt4) apoptosis

**Figure 3.**
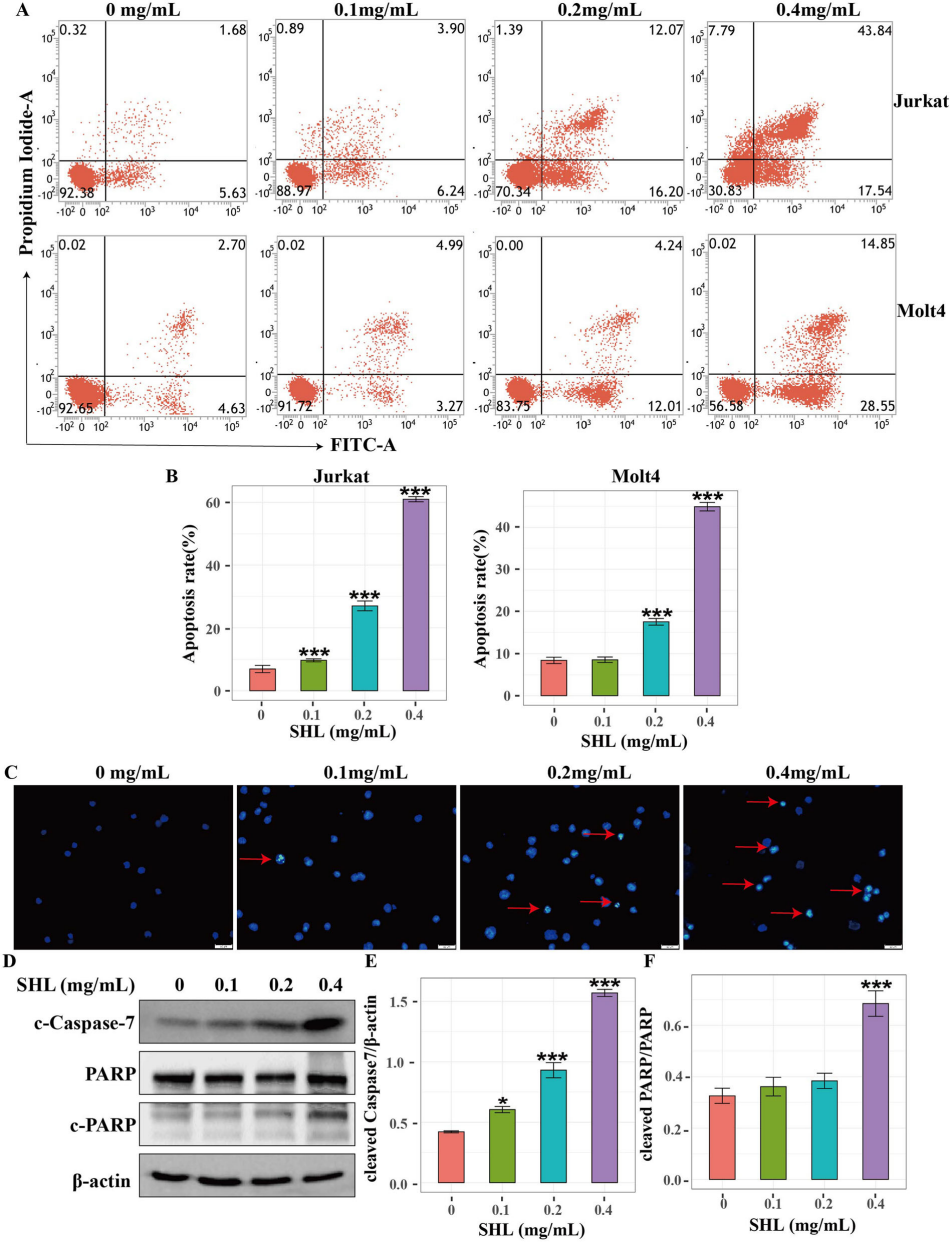
SHL induced T-ALL cell apoptosis. (A) A dot plot of Annexin V/FITC staining in Jurkat and Molt4 cells after treatment with SHL for 24 h. (B) Bar chart showing the apoptosis rates of Jurkat and Molt4 cells after treatment with SHL for 24 h. (C) Jurkat cells were treated with SHL for 24 h, after which the cells were photographed under a phase contrast microscope. The arrows indicate apoptotic cells. (D) Western blot analysis of cleaved caspase 7 and PARP in Jurkat cells after treatment with SHL. (E,F) The relative protein levels of cleaved caspase 7 and cleaved PARP in Jurkat cells. All data are expressed as the means ± SD. *p < 0.05 compared with the corresponding controls. ***p < 0.001 compared with the corresponding controls.

To confirm this hypothesis, flow cytometry was used to detect the apoptosis of T-ALL cells (Jurkat and Molt4). After treatment with different concentrations of SHL (0, 0.1, 0.2, 0.4 mg/mL) for 24 h, Jurkat and Molt4 cells were collected. The results showed that the apoptosis rate in both cell lines increased in a dose-dependent manner (Figure 3(A,B)). The apoptosis rate increased from 6.92 ± 2.00% to 61.01 ± 1.39% for Jurkat cells and 8.39 ± 1.31% to 44.91 ± 1.75% for Molt4 cells (Figure 3(A,B)). Moreover, morphological apoptosis was evaluated to reconfirm the apoptosis of Jurkat cells. As shown in Figure 3(C), an increase in the number of apoptotic cells was observed with increasing SHL concentration under fluorescence microscopy. Finally, the expression of proteins related to apoptosis was measured by Western blotting. The results showed that the level of an apoptosis executor protein, cleaved caspase7 increased in a dose-dependent manner in Jurkat cells. The cleaved PARP (cleaved poly ADP-ribose polymerase) protein level also increased with increasing SHL concentration (Figure 3(D–F)). These results showed that SHL induced apoptosis in T-ALL.

### SHL-induced apoptosis can be rescued by an apoptosis inhibitor

**Figure 4.**
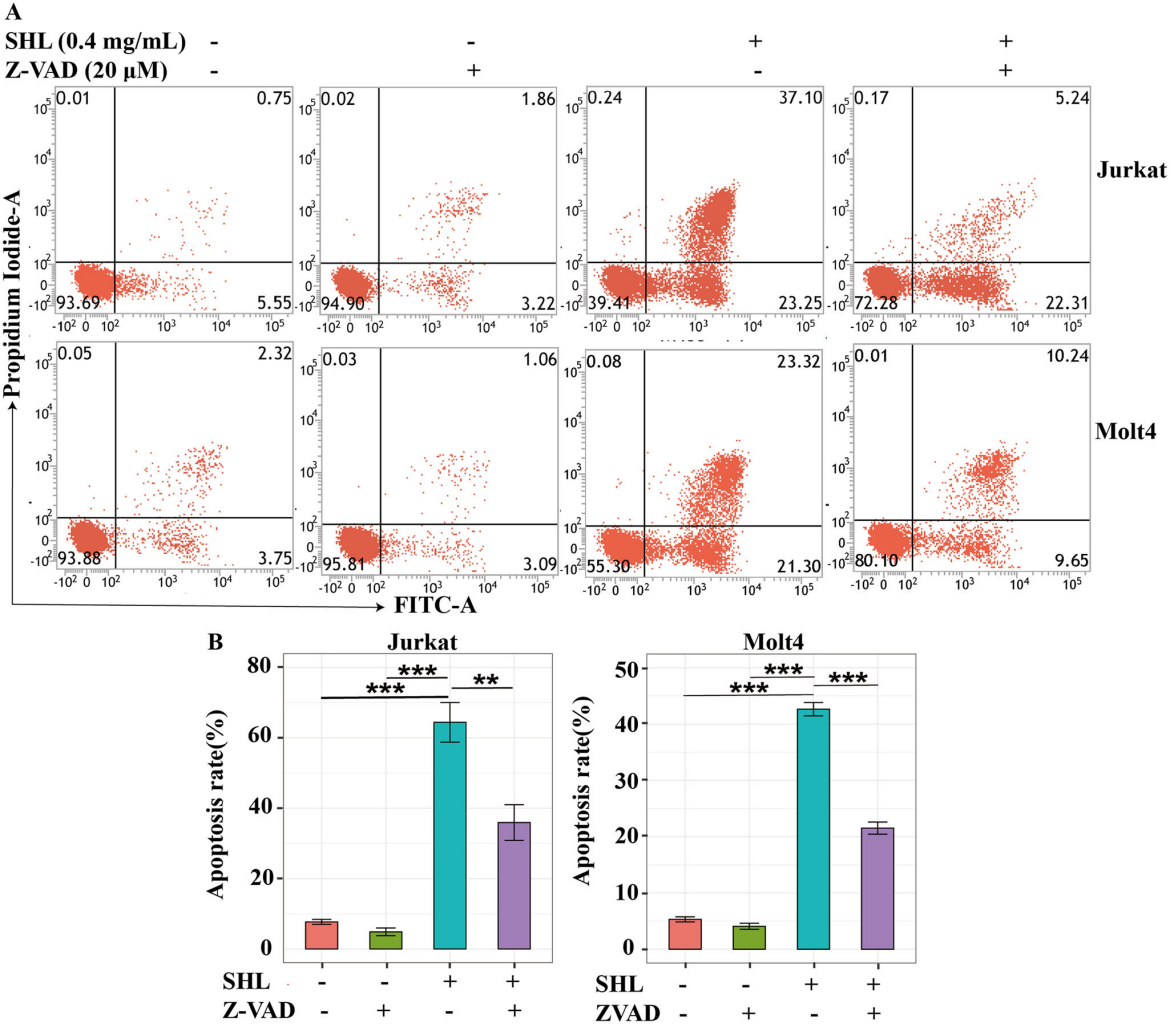
SHL-induced apoptosis can be rescued by Z-VAD-FMK. (A) A dot plot of Annexin V/FITC staining in Jurkat and Molt4 cells after treatment with SHL and Z-VAD-FMK for 24 h. (B) Bar chart showing the apoptosis rates of Jurkat and Molt4 cells after treatment with SHL and Z-VAD-FMK for 24 h. Jurkat and Molt4 cells were divided into four groups. The four groups of cells were treated with RPMI 1640 medium containing 10% foetal bovine serum, SHL (0.4 mg/mL), Z-VAD-FMK (20 μM/mL) or combination of SHL (0.4 mg/mL) and Z-VAD-FMK (20 μM/mL) for 24 h, and cells were collected for analysis. All data are expressed as the means ± SD. **p < 0.01, ***p < 0.001.

To further verify whether SHL promotes cell death through apoptosis, an apoptosis inhibitor (Z-VAD-FMK) was applied. The apoptosis rates were highest in Jurkat and Molt4 cells treated with 0.4 mg/mL SHL; thus, this concentration was selected for further experiments. For this experiment, cells were divided into 4 groups: two SHL groups (0 and 0.4 mg/mL), an apoptosis inhibitor group and an apoptosis inhibitor combined with SHL group. As shown in Figure 4, the apoptosis rates of Jurkat cells after 24 h of treatment in RPMI 1640 medium containing 10% foetal bovine serum, Z-VAD-FMK (20 μM) or SHL (0.4 mg/mL) were 7.69 ± 1.21%, 4.86 ± 1.89% and 64.40 ± 9.77%, respectively (Figure 4(B)). The apoptosis rate of Jurkat cells treated with the combination of Z-VAD-FMK (20 μM) and SHL (0.4 mg/mL) was 35.94 ± 8.79% (Figure 4(B)). After treatment with RPMI 1640 medium containing 10% foetal bovine serum, Z-VAD-FMK (20 μM) or SHL (0.4 mg/mL) separately, the apoptosis rates of Molt4 cells were 5.31 ± 0.78%, 4.07 ± 0.92% and 42.64 ± 2.07%, respectively (Figure 4(B)). After treatment with the combination of Z-VAD-FMK (20 μM) and SHL (0.4 mg/mL), the apoptosis rate of Molt4 cells was 21.51 ± 1.86% (Figure 4(B)). The apoptosis rate of Jurkat and Molt4 cells treated with SHL (0.4 mg/mL) were higher than those of cells treated with Z-VAD-FMK (20 μM) and RPMI 1640 medium containing 10% foetal bovine serum (Figure 4(A,B)). Compared with that of cells treated with SHL, the apoptosis rate of cells treated with the combination of SHL and Z-VAD-FMK was decreased (Figure 4(A,B)). These results suggested that SHL-induced T-ALL cell apoptosis can be rescued by an apoptosis inhibitor (Z-VAD-FMK).

### SHL can inhibit the NOTCH1-MYC signalling pathway

**Figure 5.**
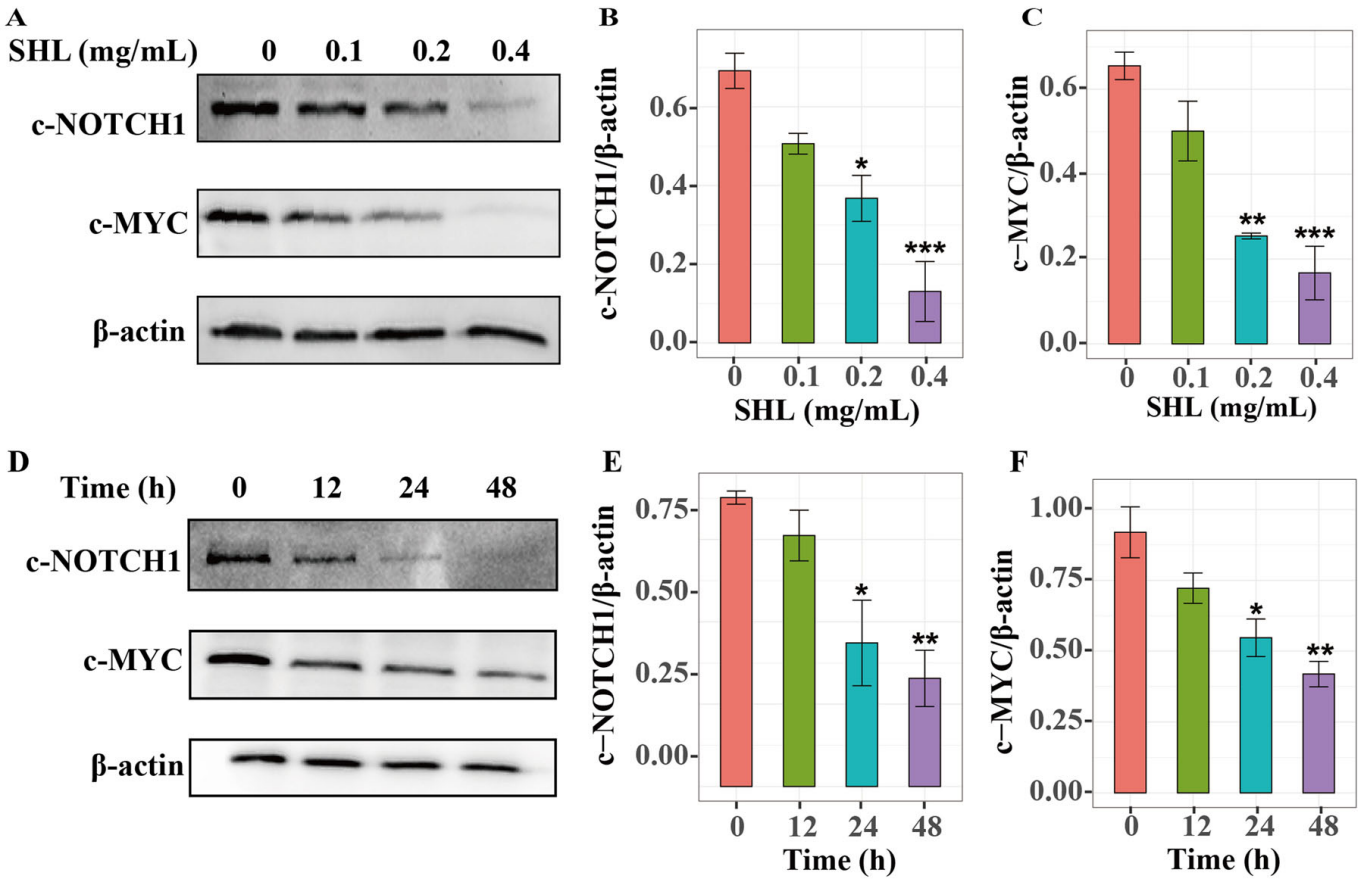
SHL can inhibit the NOTCH1-MYC signalling pathway. (A) Western blot analysis of cleaved NOTCH1 (c-NOTCH1) and c-MYC in Jurkat cells after treatment with SHL (0, 0.1, 0.2 and 0.4 mg/mL). (B,C) The relative protein levels of cleaved NOTCH1 and c-MYC. (D) Western blot analysis of cleaved NOTCH1 and c-MYC in Jurkat cells after treatment with 0.2 mg/mL SHL (0, 12, 24 and 48 h). (E,F) The relative protein levels of cleaved NOTCH1 and c-MYC. All data are expressed as the means ± SD. *p < 0.05, **p < 0.01, ***p < 0.001 compared with the corresponding controls.

To identify how SHL induces apoptosis, GO enrichment analysis was further performed with the 117 target genes. One-third of the enriched common signalling pathways in T-ALL were related to the NOTCH signalling pathway (Figure 2(C)). It has been reported that hyperactive NOTCH1 signalling plays crucial roles in the pathogenesis of T-ALL (Sanchez-Martin and Ferrando 2017). The MYC oncogene is one of the key factors in NOTCH1-induced transformation (Bray and Gomez-Lamarca 2018; Sprinzak and Blacklow 2021). We hypothesized that SHL induces T-ALL cell death through regulation of the NOTCH1-MYC signalling pathway. Therefore, Western blotting was used to verify whether SHL can inhibit the NOTCH1-MYC pathway. Compared with those in Molt4 cells, the IC_50_ values of SHL in Jurkat cells were lower, leading to a higher apoptosis rate. More importantly, based on data from the Project Achilles (Meyers et al. 2017; Shi et al. 2021), it was found that NOTCH1 plays a more important role in the survival and maintenance of Jurkat cells than of Molt4 cells (Figure S3). Therefore, Jurkat cells were selected for further validation. Jurkat cells were treated with different concentrations of SHL (0, 0.1, 0.2 and 0.4 mg/mL) for 24 h and collected to measure the protein levels of cleaved NOTCH1 and c-MYC. The cleaved NOTCH1 protein level decreased in a dose-dependent manner in Jurkat cells. Similarly, the c-MYC protein level decreased significantly with increasing SHL concentration (Figure 5(A–C)). The cleaved NOTCH1 and c-MYC protein levels showed time-dependent decreases in Jurkat cells treated with 0.2 mg/mL SHL for 0, 12, 24 and 48 h (Figure 5(D,E)). These results confirmed our hypothesis mentioned above.

### The active component of SHL can bind to γ-secretase, which directly catalyses the cleavage of NOTCH1

**Figure 6.**
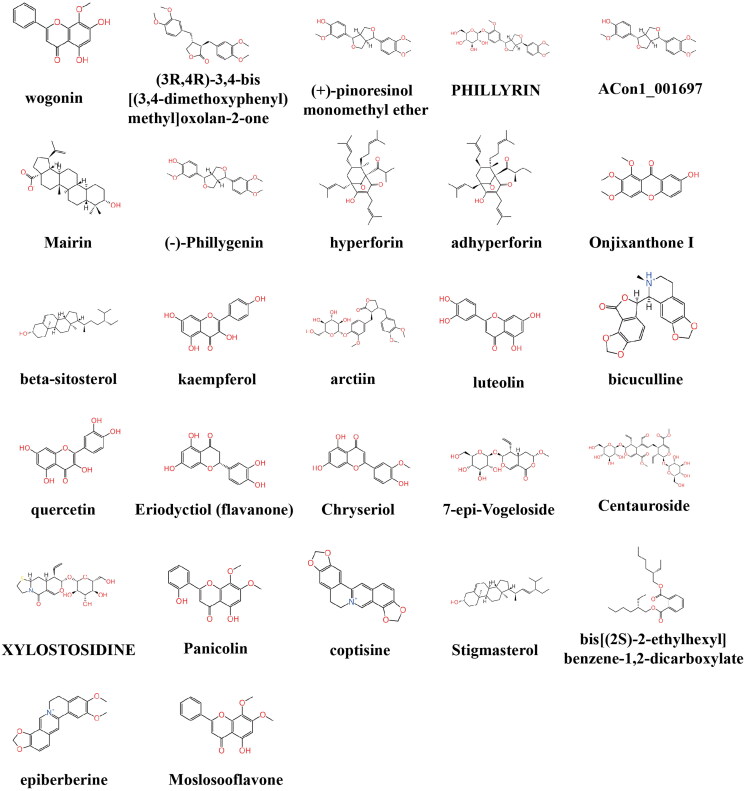
The active components of SHL.

**Figure 7.**
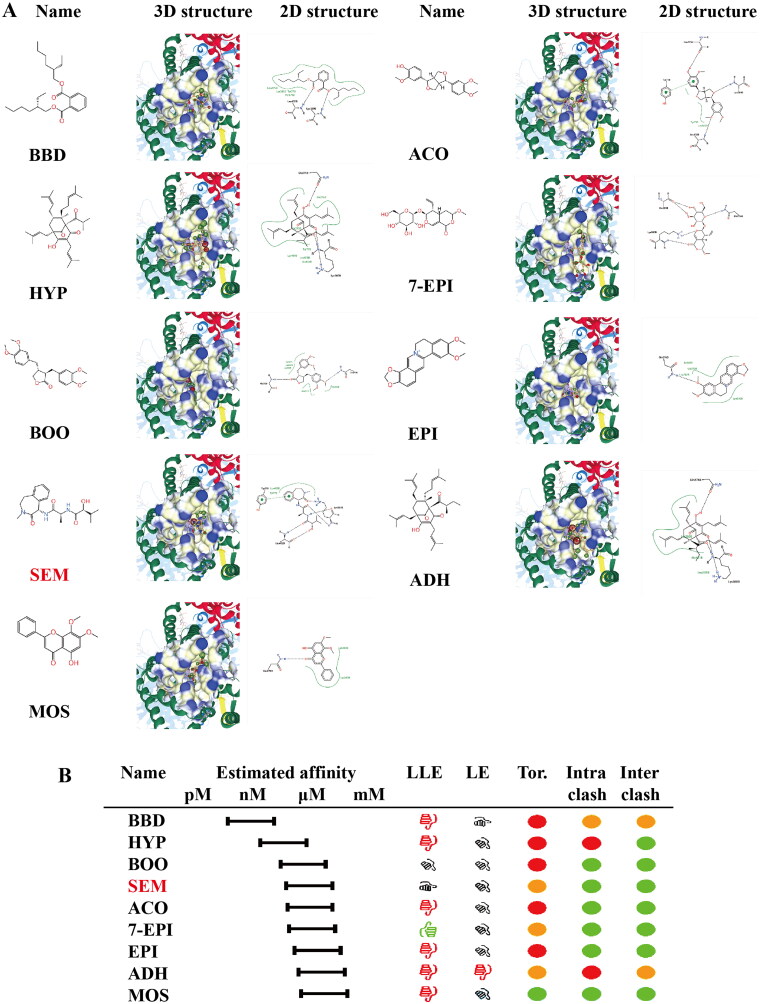
The active components of SHL can bind to γ-secretase. (A) The binding site of γ-secretase to its ligand molecule. The 2D structures of compounds with affinities below μM are displayed as well as the 2D and 3D molecular structures of the binding sites. (B) Molecular docking results of the γ-secretase ligands highlighting the estimated affinity compared to SEM. All drugs with an estimated lower bound binding affinity below 100 μM were chosen to run a screening with 70 poses. Red circle = unfavourable. Green circle = favourable. BBD: *bis*[(2*S*)-2-ethylhexyl]benzene-1,2-dicarboxylate; HYP: hyperforin; BOO: (3R,4R)-3,4-*bis*[(3,4-dimethoxyphenyl)methyl]oxolan-2-one; SEM: semagacestat; ACO: ACon1_001697; 7-EPI: 7-*epi*-vogeloside; EPI: *epi*-berberine; ADH: adhyperforin; MOS: moslosooflavone; LLE: hydrophobic ligand efficiency; LE: ligand efficiency; Tor.: torsion quality; Intra clash: intra molecular clash; Inter clash: inter molecular clash.

It is well known that c-MYC expression is promoted after the NOTCH1 protein is cleaved by γ-secretase (Bray and Gomez-Lamarca 2018; Sprinzak and Blacklow 2021). To explore whether the inhibition of the NOTCH1-MYC pathway is caused by interactions between the active components of SHL and γ-secretase, analysis of molecular docking between the active components of SHL and γ-secretase was performed. Considering the druglike properties, especially false-positive hits and the clarity of the molecular structure, the long-chain alkyl ingredients of SHL were eliminated and 27 active ingredients were retained and used for molecular docking simulation (Figure 6). Semagacestat has been reported to inhibit the activity of γ-secretase by binding to the relevant active pocket (Iraji et al. 2020). Molecular docking studies were performed on these ligands to determine their binding sites (Figure 7(A)) and affinities (Figure 7(B)). Compared with semagacestat, three kinds of ligands interacted with γ-secretase with increased affinity, and five kinds of ligands interacted with decreased affinity, indicating that the active ingredients of SHL can bind to γ-secretase. We measured the activity of γ-secretase by ELISA and found that the activity of γ-secretase decreased with increasing SHL concentration (Figure S2). Therefore, we speculated that SHL can target γ-secretase to reduce its activity and thus inhibit the NOTCH1-MYC pathway.

## Discussion

SHL is available in various dosage forms and is a typical traditional Chinese patent medicine with a long a history of application in treating respiratory tract infections in China (Su et al. 2020); thus, it is safe for use in the treatment of ALL. The antitumour effect of SHL has rarely been reported. This study showed that the lyophilized powder used for injection of SHL exhibited an anti-T-ALL effect. However, due to the complex chemical composition of SHL, it is difficult to elucidate its precise pharmacological mechanisms in the treatment of T-ALL.

In this study, we employed network pharmacology combined with molecular docking as well as experimental studies to validate the possible mechanism of SHL in T-ALL treatment. Multiple targets were identified for both T-ALL and SHL. A total of 117 overlapping targets between T-ALL and SHL were identified, indicating that SHL may play an anti-T-ALL role through these targets. In addition to dying from accidental cell death, cells may die from regulated cell death (Tang et al. 2019). The forms of regulated cell death include necroptosis, pyroptosis, ferroptosis, parthanatos, entotic cell death, netotic cell death, lysosome-dependent cell death, apoptosis, autophagy-dependent cell death, alkaliptosis and oxeiptosis (Green 2019; Ahamed et al. 2022). Through GO enrichment analysis of the 117 targets, we found that SHL mainly targeted the extrinsic apoptotic signalling pathway, intrinsic apoptotic signalling pathway, regulation of G1/S-phase transition pathway, regulation of G2/M- phase transition pathway, regulation of autophagy pathway, necrotic cell death pathway and pyroptosis pathway. Among these signalling pathways, SHL predominantly targeted the apoptosis signalling pathway. We identified 52 active ingredients of SHL through analysis. It has been reported that arctiin can induce apoptosis and potentiate bortezomib-induced apoptotic effects in human multiple myeloma cells (Lee et al. 2019). Han et al. (2018) showed kaempferol increased apoptosis in human lung cancer cells by upregulating microRNA-340. In myeloid leukaemia cells, luteolin was shown to induce apoptosis *via* pituitary tumour-transforming gene 1 (Chen et al. 2018). Quercetin was found to induce apoptosis in colorectal cancer mainly through the AMPK pathway and ERβ- dependent signalling (Darband et al. 2018). These studies with active components of SHL provide supportive evidence that SHL can induce apoptosis, consistent with our findings. After the apoptotic process is initiated, an increase in caspase-7 cleavage leads to the cleavage of PARP (Desroches and Denault 2019; Li et al. 2020). Our study also showed that the levels of cleaved caspase-7 and cleaved PARP increased with increasing concentration of SHL. More importantly, we found that SHL-induced T-ALL cell apoptosis was rescued by treatment with apoptosis inhibitors. *In vitro* experiments further confirmed the predictive performance of the network pharmacology approach, showing that SHL can induce apoptosis in T-ALL cells.

Due to the complex pathogenesis of T-ALL, its study is still in the exploratory stage. Currently, the most common molecular bases of the involved pathways include transcriptional regulation, lymphoid differentiation and development, NOTCH, Wnt/β-catenin signalling, JAK/STAT, PI3K/AKT/mTOR, TP53 and the cell cycle, RAS signalling, ABL1 signalling pathway, chromatin structure modifiers, epigenetic modulators, ribosomal dysfunction and altered expression of oncogenic miRNAs or long noncoding RNA (Adrián et al. 2018; Fattizzo et al. 2020). Through GO enrichment analysis of the 117 targets, we found that SHL mainly targeted the common molecular pathways in T-ALL, including the regulation of NOTCH signalling, NOTCH signalling, negative regulation of NOTCH signalling, Ras protein signal transduction, Wnt signalling, receptor signalling pathway *via* JAK-STAT, DNA methylation, histone modification and protein localization to chromatin pathways. One-third of the enriched common signalling pathways in T-ALL are related to the NOTCH signalling pathway. Furthermore, activating mutations in NOTCH1 are present in at least 60% of T-ALL cases; this aberration is the most prevalent oncogenic aberration in T-ALL (Anand et al. 2021). We experimentally proved that the level of activated NOTCH1 (cleaved NOTCH1) protein was reduced after SHL treatment. The key role of NOTCH1 and MYC in immature T cells and in the context of T-ALL is wired in a feed-forward transcriptional circuit in which NOTCH1 directly activates MYC expression (Sanchez-Martin and Ferrando 2017). MYC has been reported to be regulated by NOTCH1 in T-ALL through the NOTCH1-MYC enhancer (N-Me) (Belver et al. 2019). Based on these results, we demonstrated the downregulation of c-MYC protein expression after SHL treatment by Western blot analysis. *In vitro* experiments confirmed the predictive performance of our network pharmacology approach, showing that SHL can inhibit a key signalling pathway (NOTCH1 signalling pathway) in T-ALL. It has been reported that c-MYC expression is increased due to cleaved NOTCH1 produced after the substrate is cleaved by γ-secretase (Bray and Gomez-Lamarca 2018; Sprinzak and Blacklow 2021). As previously described, semagacestat is an inhibitor of γ-secretase (Iraji et al. 2020). The active ingredients of SHL could act on the same active pockets. The results indicated that SHL may have an inhibitory effect on γ-secretase similar to that of semagacestat.

Here, we reported that SHL treatment induced apoptosis and inhibited c-MYC protein expression by binding to γ-secretase and downregulating NOTCH1 cleavage. Taken together, these findings proved that SHL might be a promising therapeutic agent for T-ALL.
